# The Mirror Has Two Faces: Contradictory Reflections of Donkeys in Western Literature from Lucius to Balthazar

**DOI:** 10.3390/ani1010056

**Published:** 2010-12-14

**Authors:** Jill Bough

**Affiliations:** School of Humanities and Social Sciences, Ourimbah Campus, University of Newcastle, Newcastle, NSW 2258, Australia; E-Mail: Jill.Bough@newcastle.edu.au

**Keywords:** donkeys, cultural representations, literature

## Abstract

**Simple Summary:**

I argue that cultural representations of donkeys arise from the contradictory philosophies that underpin western society. Donkeys have invariable been used symbolically in a negative way in early western literature which has affected our ongoing attitudes towards them: used as allegories for human nature, their own remains largely hidden. Tracing literary representations of donkeys reveals not only their conflicting origins but also how they developed over time. Understanding how these representations have affected our treatment of donkeys may lead to a better appreciation of the actual animals.

**Abstract:**

How we represent animals both reflects our attitudes towards them and affects our treatment of them. The donkey has lived alongside humans, bearing their burdens since the time of their domestication over 10,000 years ago. Despite this, they have invariably enjoyed a low status in human cultures, received little appreciation and been treated harshly. We view some animals as being more worthy than others and represent them accordingly: donkeys have been ridiculed and derided. Literary representations of donkeys from the fables of Ancient Greece to contemporary iconic texts are explored to follow the donkey through the human imaginary. These representations derive from two main, conflicting sources, Greek literature and the Bible. Examining these cultural representations may lead towards a greater understanding of the way they affect the actual animal and lead to a greater understanding of that animal and, ultimately, to better treatment of them.

## 1. Introduction to Representations of Donkeys in the West

The Donkey

When fishes flew and forests walk'd

And figs grew upon thorn,

Some moment when the moon was blood

Then surely was I born;

With monstrous head and sickening cry

And ears like errant wings,

The devils walking parody

On all four- footed things

The tatter'd outlaw of the earth,

Of ancient crooked will;

Starve, scourge, deride me: I am dumb,

I keep my secret still.

Fools! For I also had my hour;

One far fierce hour and sweet:

There was a shout about my ears,

And palms before my feet [1].

The purpose of this paper is to explore the ways donkeys have been represented in a number of iconic texts in Western culture because re-examining the ways in which they have been perceived may lead to a new way of thinking about and living with donkeys [2]. Literature that features donkeys has a place in helping to determine human perceptions of, attitudes towards and actual treatment of donkeys. As Steve Baker suggests, an examination of these cultural representations may stimulate our concern for the circumstances of actual living animals in that same culture [3]. Representations of donkeys in western literature are contradictory and confusing because donkeys are invariably used as allegories for human behaviour. These representations derive from two main, conflicting sources, Ancient Greek literature and the Bible. The Greeks represented the donkey in a negative light while a more benign meaning became popular during the Middle Ages, when the donkey came to be associated with Christianity. This paper reviews a selection of literature, across time, place and genre and considers changing attitudes towards donkeys in those representations. Although the texts illustrate how donkeys are used as metaphors for humanity, I further argue here that these representations can also reveal something of what it is like to be a ‘real’ donkey [4].

The opposing representations of donkeys are significant because they illustrate the deep contradictions within the very foundations of western society: the Greco-Roman philosophy and the Jewish/Christian ideology which underpins it. The writings of Homer, Aesop and Apuleius, for example, have been instrumental in representations of donkeys as servile, stubborn and stupid, while biblical imagery has been influential in presenting donkeys as symbols of humility and peace, suffering and service. This arises particularly from the donkey's association with Jesus, Mary and the Nativity in the New Testament. Some biblical stories suggest a belief that donkeys had a special connection with the spiritual world which attests to the fact that donkeys were believed to have prophetic abilities. Perhaps the most famous of these references is the story of Balaam's ass in the Old Testament; God chose to speak through a donkey to show Balaam the error of his ways. Not only does she represent wisdom, rather than stupidity, she also exemplifies the suffering and abused beast of burden. The donkey, patiently bearing harsh abuse, as well as the physical burdens placed upon them, is often represented as a Christ-like figure. This is reinforced by Jesus’ triumphant entry into Jerusalem on the back of a donkey, one of the most enduring Christian symbols in Western culture. These differing representations, whether favourable or adverse, have been significant in subsequent representations of donkeys. Although many texts have been influenced by images of Christ and the donkey, of humility, suffering and patience, this is not necessarily how they are generally perceived, nor is it reflected in their treatment in the real world. What follows may help to explain why that is so.

## 2. Classical Greek Literature

Classical Greek literature reveals a great deal about attitudes to donkeys at the time and those representations remain with us today. Greek society was hierarchical and the employment of slaves and domestic animals integral to the functioning of that society. Especially significant has been the contrasting representation of horses and donkeys. Horses were considered magnificent beasts and the owning and breeding of horses was an indication of wealth and class. Horses are widely represented in Greek literature and art whereas there is little artistic representation of donkeys. In the ancient world, the horse represented freedom, power and beauty, qualities that humans not only valued but also desired for themselves. A sharp cultural distinction was made between the horse and the donkey which corresponded in many respects to the distinctions between the upper and lower classes in Greek society [5]. The donkey's lowly status as compared to the esteem in which the horse was held is evident in, for example, two similes in Homer's *Iliad* (Book 11). In one, the Trojan prince, Paris, is compared to a horse who, freed form his stable, gallops across the open plain, proud and strong, with mane flowing and head held high. In another, the Greek hero Ajax, in his fight against the Trojans, is compared to a donkey who has gone into a field and will not lift his head from eating grain, despite the fact that he is beaten with sticks.

Just as when some donkey taken past a cornfield—
a stubborn beast on whose sides many sticks are broken—
bolts from boys tending it and goes to munch deep corn, while boys beat it with sticks—
although their strength is small
at last they drive it out, once it's had its fill—

The donkey is thus represented as greedy, stubborn and mundane, concerned only with filling his belly, unlike the imposing and proud horse who is motivated by the finer desires of nobility, freedom and mastery. Both, however, represent the human condition. The horse represents our dreams of grace and beauty, of power and independence; unlike the servile donkey who represents our day to day, careworn and monotonous lives [6].

Despite the lack of donkeys in Greek higher literature, they are common in popular proverbs and fables, especially Aesop's *Fables* (6th Century BCE) which highlight human folly, ambition and greed, especially amongst the lower classes. Aesop alone wrote around twenty fables about ‘donkeys’ and they are invariably depicted in a negative light, as servile and lazy, selfish and stupid; used as examples of human folly and weakness. In the fable, *The Ass in the Lion's Skin* a silly donkey is very proud of himself when he puts on a lion's skin that had been left out to dry by some hunters. All fled at his approach and he felt very important. In his delight he lifted his head and brayed; everyone then recognised him and his owner gave him a sound beating. The moral is clear, not even a fine disguise will hide a fool. In another fable, a donkey who wanted to sing like a cicada asked the insect what he ate. When he learned that it was dew, the donkey ate nothing but dew and very soon died from starvation. The lower classes, represented by the dumb donkey are without aesthetic abilities; they do not dance and sing as the aristocracy does [7]. There is nothing about ‘real’ donkeys in these representations; they are used solely as symbols for human behaviour. However, it is largely from such fables that the donkey's reputation for foolishness and stupidity has been encouraged and perpetuated. Aristotle had stated that it was natural for free men to dominate slaves and animals because both were much less rational than free men and actually benefitted from domination [8]. It was therefore acceptable not only to denigrate and ridicule the lowly donkey but also to abuse them physically.

Probably the most influential story in determining attitudes to the donkey was that written in Roman Africa by Apuleius. *The Golden Ass* (160AD), a version of a fable that also survived in Greek, recounted the adventures of a young Greek called Lucius [9]. His dabbling in magic resulted in him accidentally being transformed into a donkey, not a wise owl, flying free, as he had wished [10]. Lucius' humiliating metamorphosis and subsequent experiences as he is sold from one owner to another, allowed Apuleius to construct a series of comic stories based on the characteristics commonly attributed to donkeys at that time, such as stubbornness, foolishness and lust [11]. Although it could be argued that Lucius retains his own human characteristics of unwise curiosity, a foolhardy sense of adventure and sexual license, his faults seem all the more gross within the body of an ass. Apuleius represents the donkey as both an allegory for human behaviour and as a real and suffering beast of burden: the actual life and treatment of donkeys and their symbolic status are inextricably entwined, each affecting the other. Inwardly, Lucius is a human and he can therefore think and respond to situations rationally and morally. The transformation has been only external: “although fully an ass and beast of burden instead of Lucius, I nevertheless kept human sense”. The experience of the transformation depends on the antithesis between human and animal accepted at the time. It is significant that the human soul remains within the body of this beast; conversely, many of the humans in the story act in degraded and ‘bestial’ ways. Questions are therefore raised as to what is properly human and about the accepted hierarchy of humans over animals [12].

Outwardly, Lucius is an ass; although he can think, he cannot put his thoughts into words, he can only hee-haw. As such, he experiences life as a beast of burden and their awful existence in Ancient Greece and Rome [13]. In the Ancient World, donkeys were the ubiquitous beast of burden and were vital to the economy. They carried crops and produce, hauled rock and timber, carried provisions and merchandise, transported food and equipment for the army and turned the heavy millstones to produce flour. The great physical strength and endurance of donkeys while requiring little care and food rendered them primary beasts of burden [14]. Lucius is used as donkeys were, as a pack animal and as a mount; however, the hardest work he endures is turning the millstone. He passes from one unpleasant character to another who abuse and mistreat him in various unsavoury and often cruel ways. He is severely beaten and even tortured for the sadistic pleasure of his human owners. At the climax of the story, Lucius is to provide a spectacle in the arena by copulating with a murderess before they are both put to death. Lucius manages to escape and he flees in fear for his life, but also in shame for what is expected of him. At this point, his human mind and animal body work in conjunction instead of in conflict. This leads to his restoration to human status after all the humiliation and abuse that he has suffered as a donkey [15]. Not only was it acceptable to beat and abuse donkeys in their lowly status, the patient donkey had come to represent all that was foolish, lustful and wicked; it is little wonder that they have never really recovered in human attitudes towards them.

## 3. Medieval and Renaissance Europe

*The Golden Ass* had a great impact on the way donkeys were perceived and depicted in literature over the ensuing centuries. In Mediaeval Europe, the Bestiaries divided animals into categories which represented the functions they fulfilled in human society; there was little evidence of interest in the reality of an animal's existence. They were divided into groups, depending on how they served human needs and were socially constructed as either ‘good’ or ‘bad’. Asses were typically presented as melancholy, heavy, dull, clumsy and slow-witted. It was Shakespeare especially who encouraged the derogatory use of ‘ass’: used frequently in his plays to describe stupid or clownish figures. This commonly used insult denoted an ignorant fellow, a perverse fool or a conceited dolt and its use would immediately give rise to laughter [16]. Most famously, perhaps, Shakespeare reworked a scene from *The Golden Ass* in *A Midsummer Night's Dream* (c. 1600), with Bottom's metamorphosis into an ass, except that Bottom is an unintelligent yokel, “a rude mechanical”, not an educated Greek gentleman.

Bottom thinks like an ass as well as himself and like Lucius and Homer's donkey before him, he longs for oats and hay to eat, more concerned with filling his belly than with the magical entertainments going on around him. As a stolid and unimaginative ass he is impervious to the advances of the beautiful Titania, which only adds to the humour because of the incongruity of the situation, the reversal of the accepted roles and the contrast of the earthy and the ethereal. Bottom represents both asshood and humanity. He is more sensible as an ass than he is as a human in which form his arrogance and self-delusion are paramount. Bottom as an ass reveals more common-sense and modesty. He is far from the sexual adventurer that Lucius was; he might be “mortally gross” but he is not lascivious [17]. As a human, Bottom is a likable clown, ‘a conceited dolt’ who is over-enthusiastic, egotistical and often ridiculous; as an ass he has more reliable, if uninspiring qualities. Here is the unimaginative and clumsy ass of Aesop's fables and the Bestiaries, lacking the sensitivities to appreciate the fantastical and magical world he has entered. The donkey is fit for everyday drudgery; he does not posses the inspired soul of the human, enabling him to enjoy the finer things in life.

Arguably the most famous novel that features a donkey as both animal companion and allegory for human nature is *Don Quixote* (1605). Cervantes reveals a different, more positive attitude towards donkeys, perhaps because donkeys had long been an important, often highly valued, aspect of life in Spain. In *Don Quxiote* the horse and donkey are both metaphors for the men that ride them. The Don's old horse, Rosinante and Dapple, Sancho Panza's beloved donkey, are both true companions and mentors to their respective masters, as well as reflections of their characters. As Antonio Vieira claims, the comparative psychology of the horse and donkey are faithfully portrayed in *Don Quixote*: “the arrogance and caprice of the horse and the humble steadiness of the donkey” [18]. Although the aim is to teach us more about what it means to be human, there is certainly an appreciation for the steadfast qualities of donkeys. Sancho's loyalty to the Don is mirrored by his relationship with Dapple. They are both simple and honest souls, good companions who enjoy nothing more than each others friendship. Dapple brings stability and security to his life and Sancho is at his happiest and most peaceful when he is riding his donkey. When they are parted, Sancho's joy at their reunion is evident:

Sancho ran immediately to his ass, and embraced him: ‘How hast thou done?’ cried he, ‘since I saw thee, my darling and treasure, my dear Dapple, the delight of my eyes, and my dearest companion!’ And then he stroked and slabbered him with kisses. The ass, for his part was as silent as could be [19].

In this romance of fantasy and chivalry, the delusions of grandeur of the knight are often grounded by his down to earth squire, who is closely associated with his donkey, both in terms of his inferior status and his personal characteristics. Sancho is corpulent and greedy and he can be stubborn in following his own dreams and self-interest. He is treated like a beast of burden at times; he is even beaten like one and brays like one on separate occasions. However, Sancho is largely to be admired for his integrity and loyalty. He comes to understand that he belongs in the real world that he knows, one of digging and ploughing the land and tending vines; he and his donkey together. Both are lowly in rank but find contentment and companionship together. The donkey belongs in his place, with the lowly in society, where he symbolises their poverty and inferior status. There are obvious resonances of Christ on the donkey, as a symbol of meekness and humility. As an actual donkey, Dapple is not abused or denigrated but appreciated for his steadfast donkey qualities of loyalty and patience. Here it could be argued that something of the ‘true’ natures of donkeys is starting to be revealed, albeit within an allegorical context.

Thus far, however, there has been little interest apparent in representing the nature of the ‘real’ animal. As well as donkeys representing human characteristics, there are human interpretations of the donkey as the archetypal robust and enduring beasts of burden, because it suited human purposes for them to be this way. We have learned of the harsh realities of their existence from ancient literature and something of their fortitude in withstanding harsh treatment. Donkeys are indeed, stoic and sturdy animals but they respond to kind treatment and proper care, as other animals do, human and nonhuman. Within an appropriate context, as any donkey enthusiast will tell you, donkeys are intelligent, sensitive, loyal and playful, as well as sensible, patient and hardy. However, they have many symbolic burdens to overcome before they can be understood in their own right. As we have seen, used as allegories for human nature, their own remains largely hidden and, as Avril Swinfen comments in respect to the portrayal of the donkey as clumsy and foolish: having become ridiculous in the popular view, “few people consider his noble qualities, and his decline in public esteem brought about a lack of care for his condition or circumstances, which in time led to actual physical decadence” [20]. However, attitudes towards animals as worthy of consideration in their own right gradually became more widely acceptable. This was reflected in certain literary representations, if not in real life.

## 4. Modern Texts: Poetry and Novels

Although an interest in animals and their welfare had been cause for debate amongst intellectuals for many centuries, the everyday plight of animals generally did not begin to improve until the latter part of the nineteenth century at a time when social change was an important aspect of the political agenda in Britain [21]. However, there was another narrative operating that had an important impact on how humans viewed nonhuman animals. Romanticism was concerned, among other things, with animals as both forces of nature and metaphors of human nature. In his poem *To a Young Ass* (1794) Coleridge highlights the contrasting attitudes popular within the educated elite with the ways in which donkeys were exploited and abused in the streets of Britain. To the educated elite, nonhuman animals had moved from being ‘brutes’ and ‘beasts’ to being fellow creatures, and, in a similar fashion to the attitudes of Saint Francis in the 12th century, even companions and brothers [22]. Coleridge was moved by the plight of a donkey foal whom he encountered on the grass at Jesus College. Coleridge felt a kinship with him and called him ‘brother’ as St Francis had before him [23]. He addresses the foal in his poem with the words: “Poor little foal of an oppressed race!” The poem continues to focus on the misery of the little foal and its chained and starving mother [24]. Although these donkeys are representative of the plight of all donkeys, they are also depicted as individual animals in their own right. They are not merely allegories for humanity and they arouse in one observer at least a compassion for their neglect while recollecting their ancient ties with the human world.

In his narrative poem *Peter Bell* (1819) William Wordsworth also showed compassion for the donkey [25]. The poem is based on an incident from a newspaper, in which a donkey was found beside a canal, standing guard over the body of his dead master who had fallen in. Wordsworth threads this anecdote into his long tale, emphasising the loyalty of the donkey and his determination to stay with his master, despite being beaten and abused by Peter Bell, the man who finds and steals him. Following a journey on the donkey's back, however, Peter Bell is a reformed man:

He lifts his head and sees the Ass

Yet standing in the clear moonshine;

“When shall I be as good as thou?

Oh! Would, poor beast, that I had now

A heart but half as good as thine!”

Obviously Peter Bell's journey on the donkey's back has Christian significance as does his viewing the donkey as a noble and good. The patient and loyal donkey is a symbol of Christianity, yet these are also actual donkey characteristics; they are not allegories of human failings. A different attitude to donkeys is becoming apparent with an appreciation of their ‘good hearts’ and as loyal companions, at least in some of the literature appearing at this time. This did not necessarily have a widespread effect on the way in which donkeys were treated in the real world, however, where they were abused, overworked and neglected [26].

Appreciation of her steadfast qualities were not in the forefront of Robert Louis Stevenson's mind as he described the journey he made through the Cevennes in southern France with a little mouse grey donkey in his book *Travels with a Donkey* (1879) [27]. To him, Modestine is simply a beast of burden. He constantly loses patience with their slow progress and he hits her to try to make her go faster. He has already loaded her up with a very heavy bundle and he hits her twice across the face. Her response, however, makes him stop and think: “It was pitiful to see her lift up her head with shut eyes, as if waiting for another blow. I came very near to crying”. He lightens her load—but continues to beat her and prick her flesh with a sharp goad. It is only when he has sold her and he is alone that he appreciates what she meant to him. “I had lost Modestine. Up to that moment I had thought I hated her, but now she was gone, ‘And oh the difference to me!’…” Stevenson's treatment of Modestine reflects the more usual attitude towards donkeys, as unfeeling and stubborn beasts of burden. Although he realises what he has lost once she has gone, it is in regard to his own loss, rather than an appreciation of his donkey companion. She is punished and abandoned at the whim of a thoughtless human master; a common fate for donkeys.

A totally different attitude is evident in Jimenez's relationship with his donkey companion in *Platero and I* (1914) [28]. On Jimenez' travels through Andalucía, Platero is treated as an equal and a friend. This is indeed a contemporary interpretation of human and animal interrelationships and it mirrors and reflects, albeit in a fictional and allegorical context, debates about the superiority of humans—and the lowly inferiority of donkeys. Jimenez's donkey is his companion and confidant with whom he has long, philosophical conversations. This is the human and nonhuman animal bond at its most serene. Jimenez grieves for all the donkeys that have been badly used and is distressed by the fate of all the neglected donkeys that they come across on their travels, such as the ancient donkey who has been abandoned at the rubbish tip where “he will freeze to death in this high ravine, pierced by the north wind …” Jimenez does not see the difference in status between himself and Platero and he takes his friend to visit a beautiful orchard. The guard at the entrance tells him that he may not enter with the donkey. Jimenez is surprised, “What donkey?” When he realises that the guard means his friend, Platero, they go on their way without visiting the orchard. It is an expression of human friendship bestowed on a donkey. After all, we cannot know if Platero feels as his master does about their relationship. Jimenez imagines what it is like to be a donkey and it is through such attempts that we can aspire to a more compassionate outlook. It seems to me that they are not totally anthropocentric; such attempts reveal more than what it means to be human: we learn also something about what it means to be a donkey.

Perhaps the most famous of all literary donkeys in the West, especially amongst children, is Eeyore in *Winnie the Pooh* (1926). As we have seen, donkeys as dependable companions form a popular thread in modern western literature: however, they are also invariably used as human stereotypes. In this book, the animals/toys are all representations of certain types of adults: Eeyore is the melancholy, grumbling old relative. In the bucolic and idealised setting of Hundred Acre Wood, Eeyore stands alone, quiet and introspective:

The old grey donkey, Eeyore stood by himself in a thistly corner of the Forest, his front feet well apart, his head on one side and thought about things. Sometimes he thought to himself sadly ‘why’ and sometimes he thought ‘wherefore’… and sometimes he didn't quite know what he *was* thinking [29].

He can be a good friend but he is most often self–absorbed and glum. Eeyore echoes the gloomy, stolid and unimaginative asses of the Bestiaries. He does not ask questions of his companions but tells them that everything “is all the same to me”. As with the donkey in Homer, Eeyore exemplifies that innate understanding of their mundane, often suffering lives and the stoic resignation to accept their fate. This is evident in many representations of donkeys and is memorably illustrated by Benjamin, the old donkey in *Animal Farm*.

In *Animal Farm* (1951), Orwell uses the animal fable for his political satire: the animals representing different aspects of human behaviour. However, Orwell explained that his concern for the treatment of farm animals also inspired this novel [30]. Once again, we have the allegorical representations and the actual life of donkeys used to compliment each other. The old donkey, Benjamin, is companion and loyal friend, to Boxer, the cart horse. He is a wise old donkey who shows little emotion but who understands more than the other animals. He is not taken in by the revolution but remains true to himself and to his one old friend. Like Eeyore, Benjamin knows nothing will really change. Revolutions will come and go but life will continue as before, harsh as it always was: “things never had been, nor ever could be much better or much worse—hunger, hardship, and disappointment being, so he said, the unalterable law of life” (Chapter 10) [31]. Benjamin is, of course, yet another human exemplar but his characteristics can be admired rather than reviled; and to those who appreciate donkeys, his attributes of common-sense, loyalty and wisdom along with the acceptance of his fate, ring true. It seems that Orwell had an understanding of the temperament of donkeys and of their resilience and quiet resignation. Although these allegorical tales are mainly intended to teach lessons about human nature, we also learn something of what it means to be a donkey. They make loyal companions, once their confidence has been earned—and they know what it is to be ridiculed and to feel lonely and outcast. They have learnt to accept their lowly place in the scheme of things and have little expectation of it improving. An underlying understanding of what it means to be a donkey in the human world is evident in these representations.

## 5. Film: Au Hazard Balthazar

The most powerful and moving representation of what it means to be a donkey in the human world is that portrayed in *Au Hasard Balthazar* (1966) [32]. It also encapsulates much of what has been written here about the representation of donkeys as the innocent figure of Balthazar highlights the donkey as an abused beast of burden and as a figure incorporating all the symbolic meanings that have been loaded onto the donkey's back. He is not, however, used as an allegory for human nature; he is represented as a real and suffering individual animal. This is especially powerful in the closing moments of the film as the battered old donkey lies down to die, bleeding from gunshot wounds, his final payment for a lifetime of abuse and mistreatment at the hands of humans. The tortured life of the abandoned animal draws to a close, bearing the burdens of human sin on his back. These are not just metaphorical burdens: he is swathed in the bags of contraband that he was forced to carry on his final journey over the Alps. The stirring sounds of Schubert's piano sonata (number 20 in A major), which opened the film with the carefree, young Balthazar, serve to heighten the tragedy of his lonely death in an Alpine meadow encircled by a flock of wandering sheep. We are left in little doubt as to the religious symbolism of the role of Balthazar in this film as he is baptised at the start of the film, is crowned with a wreath of wild flowers, carries human burdens, and is bound, harnessed and whipped by them. Although Balthazar has obvious symbolic significance in this film, he is also a real donkey who experiences pain and death. The final shot of his dead body reinforces this reality.

Although we are observers of this donkey's life, Bresson manages to convey the point of view of Balthazar in this film. There are many medium and close up shots of Balthazar's eyes or, at least, eye. Because donkeys have eyes on the sides of their head, the shots are always side-on shots and it is these especially that suggest an inner life for Balthazar. Human representation can only be based on what humans *assume* may be an animal's experience of the world. Any representation can only be a partial understanding: it can only go as far as the human imagination allows. But this is no anthropomorphic donkey with human features and language: he is a ‘real’ donkey [33]. We can't know what a donkey is thinking but Bresson's skilful use of editing leads us to believe that we do. When Balthazar is used in a circus to cart hay to the various animals in their cages, there are point-of-view shots between the eye of the donkey and that of the other animal as they stare silently at each other. The donkey stands silently observing, he does not have easily identifiable reactions as a human actor would, but he is given an inscrutable air of mournful understanding and acceptance by the juxtaposition of shots and careful editing. We feel that we glimpse the inner life of this donkey and his communication with other animals, both human and nonhuman.

There are also many close-up and medium shots of Balthazar's legs as he ages throughout the film, representing his life's journey. In the early scenes, he has the dainty legs and tiny hooves of a little donkey foal as he gambols with the children. Later, we see those legs, now sturdy and strong, yet still so vulnerable, gallantly pulling loads or bearing people, sometime trotting briskly as he clatters along the road. At others, he is driven too fast or pulls too great a load and we see those legs struggling to move forward or stumbling over uneven and rocky terrain. As with Lucius, it is turning the millstone which is the most gruelling task he is forced to perform. He is now older and weaker, the work tedious and hard; his legs give up on him and he stumbles, only to be beaten to make him go on. He stoically continues to do what he is programmed to do, serve his human masters. Balthazar is often made fun of because he is an anachronism in a modern world but we observe his patience and nobility in the face of inadequate and selfish humans.

As a narrative, *Balthazar* borrows from a number of the literary traditions already mentioned. It is a Christian allegory, a fable and a parable of sin and suffering. It is inspired in parts by *The Golden Ass* by Apuleius and *The Idiot* by Dostoevsky [34]. The structure of Balthazar's story is similar to that of *The Golden Ass* as he is passed from person to person. Balthazar is pure and good; he meekly accepts his fate because he must; he has no control over what happens to him. Bresson, who has a high regard for donkeys, is well-aware of their life when viewed simply as a beast of burden He has said about Balthazar that he is simply a donkey who:

walks or waits, regarding everything with the clarity of a donkey who knows it is a beast of burden, and that its life consists of either bearing or not bearing, of feeling pain or not feeling pain, or even feeling pleasure. All of these things are equally beyond its control [35].

Although the story of Balthazar could be considered a fable, it cannot be reduced to a simple moral. Rather than the animal being used to represent human failings, it is the humans in this tale who embody all the vices. Bresson has taken the slurs heaped on the donkey in the *The Golden Ass* and has turned the tables on human notions of their own superiority. It is the people in this story who are driven by pride, greed and lust, not the innocent donkey. He is also a real and suffering animal and he typifies what donkeys have had to endure down the ages. Balthazar is essentially a passive character, so we learn more about his endurance and suffering in the face of adversity than we do about other donkey characteristics; however, his loyalty and longing for affection is made evident. Christian symbolism and the life experiences of this donkey combine powerfully in Bresson's representation to raise awareness and compassion for donkeys. The wickednesses and weaknesses are unloaded from the back of Lucius and placed firmly at the feet of the humans in this film. We are faced with our own human failings instead of attributing them to the donkey. We are also asked to question the treatment meted out to this donkey: indeed, to all of his kind throughout human history and to rethink out our attitudes towards them.

There is growing acceptance that we need to rethink our relationship with animals. An important aspect of that awareness is an examination of a society's cultural representations of animals. Any form of representation may help to educate us about how society treats the subject of that art. The donkey continues to be exploited and used as symbol, for whatever purposes humans choose, whether stupidity or simplicity, gentleness or strength, humility or humour, stubbornness or endurance, loyalty or laziness. We need to rethink our assumed position of superiority and our condescending attitude. A film such as *Au Hasard Balthazar* confronts us with the truth of the animal's inherent goodness compared with the weakness and wickedness of humans. Bresson takes us on a donkey's journey through the harsh realities of a cruel world and we cannot help but empathise with Balthazar and his kind. As Randy Malamud maintains, wanting to discover what it is like to be an animal is the beginning of actually achieving that knowledge [36]. This fosters an enlightened interaction with them and an appreciation of their significance in the world we share. That may be on our terms in our world for a domestic animal such as a donkey but we can learn a greater appreciation of and respect for their integrity and treat them with compassion.

The best spectator on the world stage is the ass

A peaceful, wise animal that feigns stupidity

But he's patient, smarter than we are

In the cool, calm way that he watches

The making and progress of history

Armies march past him and flags change

Along with the birds painted on them

While he watches unchanged.

Mahmoud Darwish [37]
